# Practical Remarks on Strabismus, with Some Novel Suggestions Respecting the Operation for Its Relief

**Published:** 1857-03

**Authors:** Thomas Graham

**Affiliations:** Late of Sydney, Australia


ART. I.—*Practical Remarks on Strabismus, with some Novel Suggestions respecting the Operation for its Relief.* By THOMAS GRAHAM, M.D., F.R.C.S., &c. &c., late of Sydney, Australia.

IT is now about seventeen years since the first operation for Strabismus, so philosophically suggested by Stromeyer, and so boldly executed by Dieffenbach, was performed. It was hailed as a great discovery, and excited an unusual amount of eagerness on the part of some of the members of our profession; and persons afflicted with this deformity became suddenly objects of interest and even solicitation.

Thousands have been operated upon, with various results; but it still admits of doubt whether the aggregate symmetry of those afflicted with this deformity has been increased, and whether the impression left upon the public mind, and even on that of the profession, is not rather unfavorable to this operation. I think it will be both interesting and instructive to the members of the profession, to examine how far there exists any legitimate ground for these unfavorable impressions, and how far the present defects of the operation admit of remedy. Very soon after the importation of this novel and ingenious proceeding into England and this country, the literature on the subject was suddenly and copiously supplied; but it was evident that some wrote rather *for* than *from* practice, and that in every case the experience was too limited, the time for observing subsequent results too short, and the mental bias in its favor too strong, to admit of a calm and satisfactory verdict upon the merits of the operation. Now that many years have elapsed, and vast numbers have been operated upon; that enthusiasm has cooled down and comparatively few cases remain, we are able to approach the subject in a more philosophic spirit, and fairly and impartially to discuss its merits and demerits. As my object in this article is chiefly practical, I do not propose to travel over the difficult and often-discussed question of the pathology of strabismus, but only to glance briefly at a few points that seem to bear upon its treatment.

Much has been said about the importance of determining which is the defective eye in any given case, and rules have been laid down for ascertaining this point. But there can be hardly any doubt, that in almost every case both eyes are equally implicated in the abnormal position; for although one may be habitually inverted and the other straight, yet if the strabismic eye be brought into play, it assumes a normal condition, and moves in obedience to the will; and the other eye, if suddenly uncovered, will be found precisely in the position that the diseased eye usually assumes; and I have proved over and over again, that as favorable a result is obtained by operating upon one as the other. The fact is, that in strabismus the two eyes start from different points; both respond to the efforts of the will, and each is found, when examined separately, to move equally well in every direction. The disease, therefore, is rather relative than positive. Practically, we should endeavor to find out which is the eye that is habitually inverted, and for this purpose should test the relative power of vision in the two eyes, and then select for operation the one in which the sight is defective. In the alternating form of the disease it is quite immaterial which is chosen, and in any case the result would be the same as regards the removal of the deformity; but the defective eye is selected in the hope of benefiting its vision simultaneously with its position.

The first point that impresses itself upon the mind of a careful observer, is the extreme variety of causes to which the disease is attributed; but when these are analyzed and grouped, they may be arranged under three heads: 1st, where the nerves supplying the muscles of the eye are affected through the brain and spinal cord, as in cases following convulsions, fevers, &c.; 2d, where irritation is propagated from the extremities of nerves, as in cases following injuries, ophthalmia, &c.; 3d, from morbid volition, as in cases resulting from temporary excitement, &c. In investigating the causes of strabismus, we find a very close analogy between this disease and talipes. Another circumstance, equally conspicuous and more embarrassing, is the numberless shades of difference in the degree of departure from the normal position, whether it be inversion, or, more rarely, eversion; or, still more rarely, elevation or depression. In extreme cases we find that when one eye occupies a central position, the other is so far drawn into the corner that about half the cornea is concealed by the folds of the caruncle. In slight cases, the departure from the central axis is from half a line to a line, and between these two extremes there is every shade of difference. The same may be said respecting divergent strabismus; but the third variety, viz., that in which either the superior or inferior straight muscle is involved, is never found in an extreme degree. Again, it is found that the condition of the sight is very different in cases that, in other respects, are apparently similar. The rule certainly is, that in cases of habitual strabismus of some years' standing, the function of the organ is impaired; but I have met with several well-marked cases in which sight has remained perfectly good, and with others in which the degree of impairment of vision has varied considerably, some being able to read large print, while others were scarcely able to distinguish features, or even large objects. In the alternating variety, vision is almost invariably found to be equally good in both eyes: and these varieties exist without any obvious change in the appearance of the eye, either as regards the condition of the pupil, or of any of the transparent media, such as may generally be observed in amaurotic cases. It is quite possible that the ophthalmoscope would reveal changes in the retina and choroid, in some of these cases, but I have not as yet had opportunities of observing a sufficient number to speak with any certainty upon this point. It has been said that the power of the external rectus muscle, and consequently of eversion, varies considerably, as evinced by the extent to which the eye can be acted upon by this muscle; and this may be true in some cases, but the statement must be received with much caution, particularly where the strabismus is extreme; because the limit to eversion of one eye depends upon the other having reached its extreme boundary.

In considering the question of treatment, I propose to limit my remarks exclusively to operative proceedings, never having been able to derive the slightest advantage from any other plan, after deformity has existed for some time. The operation that has usually been practised for the removal of this deformity has consisted in the free division of the parts attached to the inner surface of the globe, including the conjunctiva, sub-conjunctival or ocular fascia, rectus muscle and its sheath; in fact, it has been recommended by one author, that the inner surface of the sclerotic should be *cleaned.* If we bear in mind that this operation has been performed in some thousands of cases, of all ages, and presenting the numerous varieties to which I have already alluded, the wonder is, that a greater number of failures have not occurred, and that a uniform procedure should have been found so extensively applicable to so many different degrees of the deformity under consideration. When the eye is freed from the muscle, it seems in many cases to have an inherent power of assuming the straight position, so that what seems improbable in theory exists in fact. At the same time, untoward results sometimes follow the operation, either immediately or at a subsequent period, rendering the condition of the patient worse than before. As, therefore, the motives for recommending such an operation are mainly based on considerations respecting personal appearance (the improvement to sight being secondary and uncertain), it becomes a matter of great practical moment to consider what are the defects of this operation, and how far they admit of correction. The first point that strikes every close observer, even in the most favorable cases, where the eye has assumed a perfectly normal position, and moves freely, is a certain sinking in and loss of the caruncle, so that the inner part of the globe seems more exposed than that of the opposite eye, and a fossa exists in the place of the caruncle. This, so far as my experience goes, is an invariable result, and explains a circumstance that has been often remarked, that those cases are the most successful in which it has been necessary to operate upon both eyes, the double defect being less conspicuous than when one eye only has been operated upon, and is brought into comparison with the natural state of the parts upon the opposite side. Another unfavorable result that sometimes occurs, is increased prominence of the globe; this takes place usually immediately after the muscle is divided, but I have known it to occur at a subsequent period, and gradually increase for a time. This, no doubt, arises from the loss of balance of power between the straight and oblique muscles, the latter acting with undue power when one of the former is divided. It is difficult to explain why this occurs in some cases and not in others, and to indicate any sign by which such a result may be predicted; but this I shall endeavor to do in a subsequent part of this paper. The most serious and damaging effect, is the occurrence of eversion: this may be an immediate or a very remote consequence of the operation; and when the previous inversion has been slight, the increased prominence and extensive reaction in the outward direction, is sometimes very great, the deformity presenting an almost hideous appearance; to the patient it becomes a serious grievance, and to the operator a kind of haunting spectral vision. I have met with cases in my own practice, and in that of others, in which the two cornea have occupied the outer angles of the lids, particularly where both eyes have been operated upon at the same time.

There are some other minor objections to which I may briefly allude. The extensive wound in the conjunctiva heals very slowly, remains red and swollen for a considerable time, often gives rise to a fleshy growth that requires removal, and leaves a scar more or less distinct; and the power of moving the eye in the direction of the divided muscle is often quite lost, constituting in itself some deformity. In stating therefore the objections to the old operation, it may be said, that there are invariably sinking and loss of the caruncle, a scar, usually a considerable loss of power of inversion, not unfrequently an increased prominence of the globe, and occasionally eversion, more or less complete, either immediately or as a remote effect; and both prominence and eversion may coexist. Now, considering that the operation is undertaken and submitted to almost exclusively with the object of removing a deformity, it becomes a question whether this deformity is removed to a sufficient extent, and in a sufficient number of cases, to justify the proceeding; and whether one decided case of eversion does not outweigh a large number of what are usually deemed successful. Whatever may be the opinion of the profession on this point, I think it must be admitted, that, with such defects, a wide margin is left for improvement; and that if a procedure can be suggested, in which no sinking in of the caruncle occurs, nor any perceptible scar remains; in which increased prominence and eversion never take place; and in which the healing process is complete in a week, and is never attended with the formation of granulations, I think all must admit that a very important point will be gained. Such a plan I have recently been in the habit of adopting, and it is chiefly with the view of setting forth its details that I have been induced to bring the subject before the notice of the profession.

The essential principle of the operation I am about to describe, consists in the division of the muscle subconjunctivally. This, it will be remarked, is not altogether a novel suggestion; it has been recommended by M. Guerin, and has been attempted with more or less success by several surgeons in England; but it has been found difficult, and sometimes impracticable in consequence of a defective method of procedure. Thus it is suggested to draw the eye forcibly outwards, so as to render the internal rectus tense; then to introduce a small bistoury beneath the muscle and divide it. Any one, who has attempted the operation in this way, will agree with me that it is one of extreme difficulty; the loose capsule round the muscle prevents the edge of the knife from acting upon the tendon, nor can the latter be made sufficiently tense to be thus divided. The difficulty and uncertainty of this operation have resulted in its having been rarely attempted, more rarely accomplished, and *never repeated* by the same surgeon. The method that I propose, and that has been performed by myself and some of my medical friends in Sydney, Australia, in a large number of cases, is the following: Having placed the patient, if nervous or restless, or very young, under the influence of chloroform, the eyelids fixed open with a spring speculum, and the globe everted by an assistant seizing the conjunctiva near the outer margin of the cornea with a pair of forceps, the operator pinches up the conjunctiva at a point corresponding to the lower border of the internal rectus, and makes a small opening with a pair of rather strong, blunt-pointed scissors. He then seizes the subconjunctival fascia, and divides it to the same extent, so as to clearly and cleanly expose a small surface of sclerotic. The ordinary strabismus blunt hook, bent at right angles, is now made to sweep round the globe, so as to pass beneath the muscle; this requires care, and a little practice is essential to success, and may be known to be accomplished by the peculiar elastic resistance that is felt: the blades of the scissors are next passed through the opening, and by a succession of small cuts the tendon may be readily divided between the hook and the insertion into the sclerotic, and close to the latter; the clipping of the tendon may sometimes be recognized by the peculiar creaking sensation imparted to the scissors. Some little difficulty in making a complete division is experienced when the insertion of the tendon is rather broad, in which case, I am in the habit of making a small counter-opening in the conjunctiva corresponding to the upper border of the muscle, introducing the scissors from above, and having passed one blade beneath the remaining slip of tendon, dividing it in the same direction. This counter-opening has the advantage of facilitating the escape of blood that may have become infiltrated beneath the conjunctiva, and does not in any way interfere with the principle and aim of the operation, which is to leave a broad band of conjunctiva between the cornea and the inner caruncle intact. The advantages of this plan, as contrasted with the old one, seem to me to be very great. It has, in the first place, the merit possessed by all subcutaneous sections, of immunity from much subsequent inflammation and suppuration, and is, therefore, followed by a very rapid and certain cure; no granulations ever form, and the caruncle maintains its natural position, and does not shrink away into a deep fossa, as is invariably the case when the usual operation has been performed. I may also state, that, as far as my experience yet goes, increased prominence of the eye is more rare, eversion never occurs, and the natural movements of the eye are more complete. This I attribute to the fact, that the ocular fascia is but little interfered with, and that a good firm union takes place between the divided muscle and the globe of the eye.

Such seem to me to be the advantages of the operative procedure that I here recommend—advantages that are of so important a nature that, in fairly stating the case to the patient, if the old operation is contemplated, it certainly admits of doubt if the personal appearance will be much improved, even under the most favorable results; and there is always a risk of increased prominence and of eversion. If, on the other hand, my mode of procedure be adopted, the surgeon may at least feel assured, that if the deformity be not altogether removed, it will not be rendered worse; and in many cases the result will be so perfect, that the most experienced eye will not detect any defect, or be aware that any operation has been performed.

But it may be asked if there are any objections to this operation, and any cases in which the old operation is preferable. It must be admitted that it is rather more difficult to perform, that there is a greater liability to leave some portion of the tendon undivided, and that sometimes some inversion remains, in consequence of the attachment of the muscle to the fascia after it is divided from the sclerotic. This, however, will often rectify itself afterwards, and where this is not the case it is better either to operate on the other eye, or, if the cast is slight, be content to leave the case in that state, rather than risk eversion by further interference. It is only in cases of long standing, and where the strabismus is very extreme, and where the eye is small and deep-set, and where the subconjunctival operation produces but very little benefit, that the old operation is justifiable.

Before concluding this paper, I will take the opportunity to glance at two or three points of some practical interest, having reference to the age of the patient and condition of the eye, when an operation is contemplated, and at the effect of the operation upon vision.

As regards the first point, my own experience, derived from more than a hundred cases, is, that the most favorable results occur in young adults, as contrasted with children. Cases in which the relative position of the eyes is not uniform, but aggravated and altered by mental or bodily excitement, and in which there are oscillatory movements, are all uncertain in their results, and very liable to be followed by undue prominence of the globe, or by eversion, or both. On this account, I do not find operations on children succeed so well as on young adults. As regards the effect of the extent of the distortion in influencing the result of the operation, it is difficult to lay down any fixed rules; but, judging from my own experience, I should say that in extreme cases of inversion, there is only a partial improvement after the division of one muscle, and it is a nice point to determine if there be sufficient distortion remaining to make it safe to divide the inner rectus of the other eye without risking eversion. Unless the eye occupies a position at least midway between the inner canthus and the central axis, it is not safe to operate.

The chief causes of eversion after the operation, are to be traced to the previous *slight* degree of the inversion, to the unsettled state of the disease, or to an undue division and separation of parts; and if the slightest eversion occur at the time of the operation, there seems to be a constant tendency to increase until it has reached its extreme limits. This arises from the disadvantage at which union takes place, the power of the external rectus muscle, and the loss of the rectifying and controlling power of vision. Eversion sometimes occurs, weeks and months after the operation, in consequence of the gradual stretching of the uniting medium, analogous to that which sometimes takes place after fracture of the patella, in which very extensive separation of the two portions occurs. The very worst cases of eversion that I have ever seen, have resulted from a simultaneous division of the muscle of both eyes, a proceeding that is in no case justifiable. The effect of the operation upon vision is surrounded with obscurity and difficulty. In the first place, much variety exists in the extent to which vision is impaired by strabismus. In the alternating form, both eyes are equally good; in children, but little damage is done; but where the deformity has existed several years, there is almost invariably imperfect vision, differing, however, in degree in nearly every case.

In several remarkable cases that have come under my observation, as well as others under that of my friend Mr. Dixon, of London, a very sudden and complete restoration of sight has followed the operation. I should hesitate to assert this curious and almost inexplicable phenomenon, had I not verified the fact over and over again in a manner that admits of no doubt. Mr. Holthouse, of Westminster Hospital, London, has endeavored to explain this by supposing that the muscles compress the eyeball, and that the operation restores this power. If this were the true solution, we should find more uniformity in the result, but this is not the case; the improvement is sometimes gradual; and at other times no perceptible change occurs. These considerations suggest the performance of the operation during childhood, and if the result could be made equally favorable in other respects, this would be the most desirable period for its performance, the subconjunctival method rendering the usual objections to an operation at this period of life less obvious.

It will be seen that my chief object has been to describe and set forth the advantages of the subconjunctival operation; and it may be thought by some to have the disadvantage of difficulty and uncertainty, without sufficiently counteracting advantages, particularly as it is alluded to very briefly and somewhat disparagingly by recent writers on this subject, such as McKenzie, Walton, and Holthouse. After having tried it in above a hundred cases, I never now adopt the old method, and I am strongly impressed by the uniformity of a favorable result. In no case have I witnessed increased prominence or eversion. In some cases, it is true, some amount of inversion has remained, but this occurs nearly as frequently after the old operation; so that a patient may be now sure of improvement from the former method, without risking the occurrence of any of the unfavorable concomitants of the latter.

As I have mentioned in the foregoing remarks, cases of eversion following the operation of dividing the internal rectus muscle sometimes come under our observation; and as this is a very distressing deformity, far worse indeed than that for which the operation was originally performed, it may not be out of place to mention here that I have operated upon three such cases with most satisfactory results. The procedure adopted in these cases, I will briefly describe, premising, however, by stating, that it is rather difficult and tedious; should, as a general rule, be performed with the patient under the influence of chloroform; and depends mainly for its success upon a careful attention to minute details.

The eyelids being kept apart by means of the wire speculum, the structures covering the ball of the eye at the inner canthus, including the conjunctiva, cicatrix, ocular fascia, and muscle, should be all carefully dissected *en masse* from the sclerotic, in the form of a flap, the primary incision being made vertically about three lines from the margin of the cornea. Not less than one-third of the globe of the eye should be in this way exposed, which may be readily accomplished by means of scissors and forceps. This having been done, the operator should turn his attention to the external rectus muscle, which may be divided in the usual way. It is preferable that this latter should not be done previous to the dissection of the parts at the internal canthus, for the integrity of the external rectus aids very materially in steadying the eye during the performance of the operation.

The flap dissected from the internal surface of the eye is now to be stitched by means of two or three sutures to the little slip of conjunctiva left near the border of the cornea. For this purpose delicate curved needles should be employed, armed with fine silk ligatures; in their introduction the flap should be drawn forwards and downwards with the forceps, and the needles passed from within outward, entering them as near the inner canthus as possible, and bringing them out deep by the side of the ball. The requisite number of ligatures having been thus passed through the flap, the needles should then be made to penetrate the remnant of conjunctiva next the cornea, a procedure not always easily accomplished, owing to the thinness of the membrane in this situation, and the liability of the sutures to tear out. To obviate this difficulty, I make use of the following expedient: the slip of conjunctiva is dissected up toward the cornea, the needle is passed through its base, then back again so as to include a portion of the membrane, and then closely drawn. The sutures are now ready to be tied, but before doing so, as much of the larger flap as can be spared should be trimmed off with the scissors, leaving only sufficient to insure the retention of the ligatures. The latter may now be tied, the immediate effect of which ought to be a slight inversion of the eye, and it is desirable that the parts should unite in this position, for the subsequent tendency to eversion will usually correct this defect. The sutures may be allowed to remain until they ulcerate out, the inflammation produced by their presence being usually slight. The mobility of the eye is much diminished by the operation; if it can thus be made to occupy a central position, the restricted movement is a minor consideration.

I saw Mr. Bowman perform this operation at the Ophthalmic Hospital, Charing Cross, London, with his usual neatness and dexterity, and with the most satisfactory result; and having now had some personal experience in the matter, I am convinced of its practicability and successful application. It need hardly be stated, however, that its performance requires great patience and perseverance upon the part of both operator and patient, and it not unfrequently happens that it has to be repeated before the desired result is obtained. I may mention in addition, that a most satisfactory circumstance connected with a cure produced in this way, is the drawing forward of the caruncle to its natural position, thus remedying the deformity resulting from the sinking in of this little body.

